# Supplementary material for: Poor Decision Making and Sociability Impairment Following Central Serotonin Reduction in Inducible TPH2-Knockdown Rats
Source: Int J Mol Sci. 2024 May 3;25(9):5003. doi: 10.3390/ijms25095003 (PMC11084943; doi:10.3390/ijms25095003)
Supplement: Supplementary file 1 [file ijms-25-05003-s001.zip › ijms-2931414-supplementary.pdf]

Supplementary figures

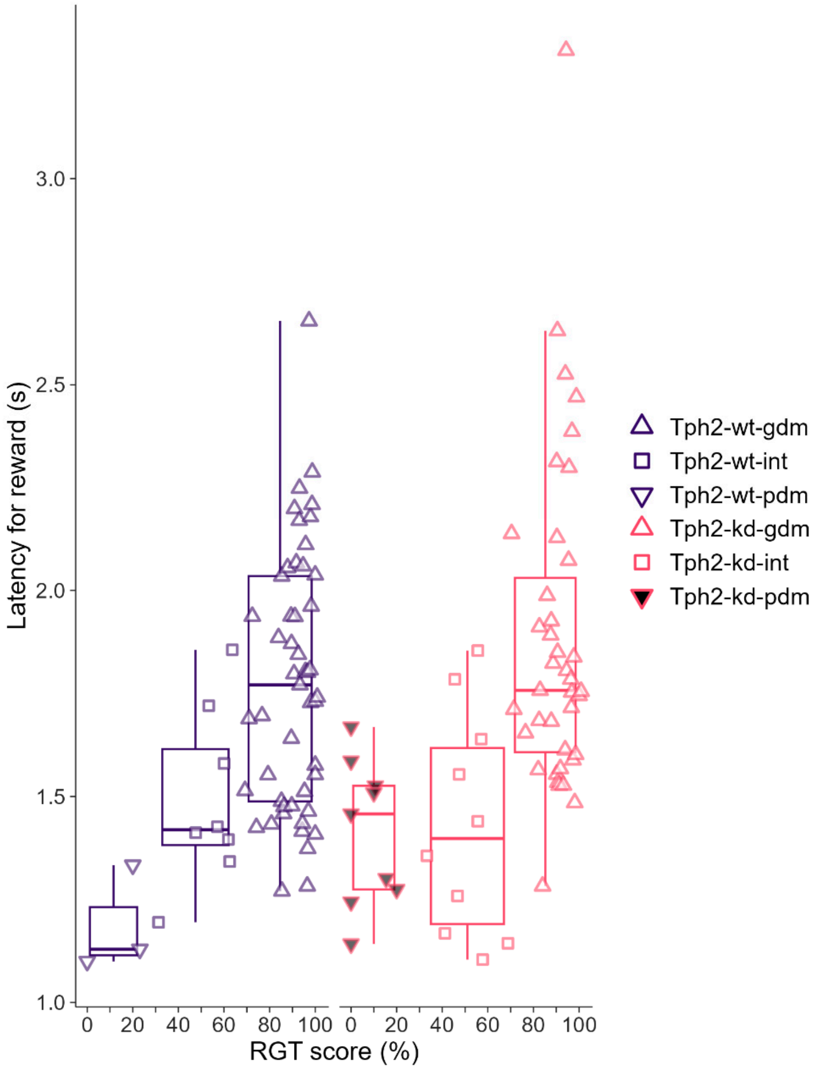

Figure S1: Latency to collect reward in RGT per decision making group and treatment groups. Good decision makers (GDMs, upward triangle), intermediates (INTs, square), and poor decision makers (PDMs, downward triangle). Individual data over boxplots. Tph2-wt in purple and Tph2-kd in pink. Tph2-wt, n = 60 (gdm, n = 49, int, n = 8, pdm, n = 3), Tph2-kd, n = 58 (gdm, n = 39, int, n = 10, pdm, n = 9), Tph2-kd n = 58 (gdm, n = 39, int, n = 10, pdm, n = 9), Tph2-kd, n = 58 (gdm, n = 39, int, n = 10, pdm, n = 9).

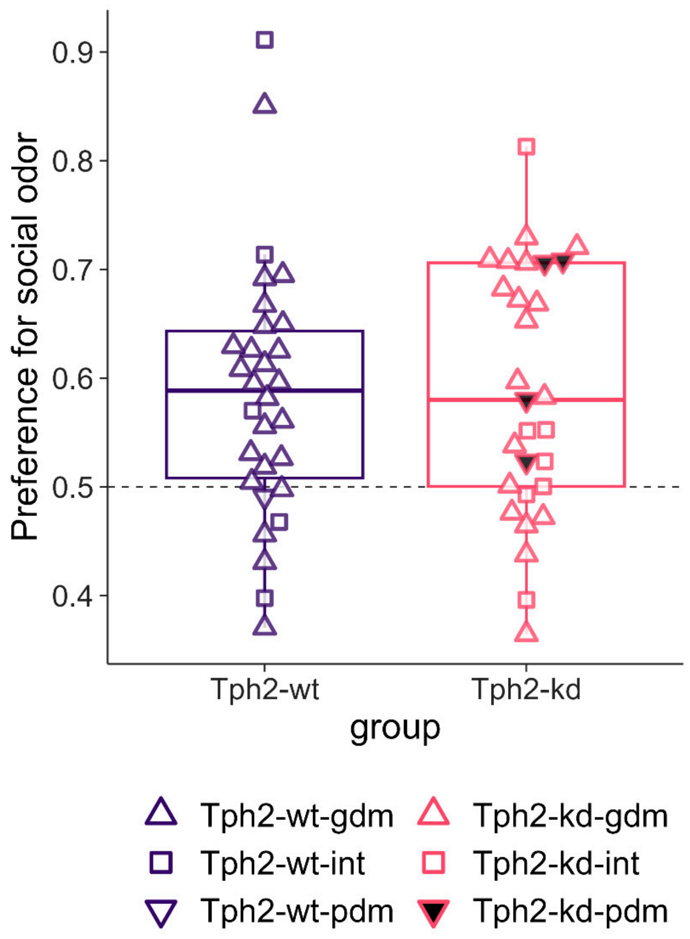

Figure S2: Odor preference per decision making group and treatment groups. Preference for the plate with the social odor. Good decision makers (GDMs, upward triangle), intermediates (INTs, square), and poor decision makers (PDMs, downward triangle). Individual data over boxplots. Tph2-wt in purple and Tph2-kd in pink. Tph2-wt n = 30, Tph2-kd n = 29.

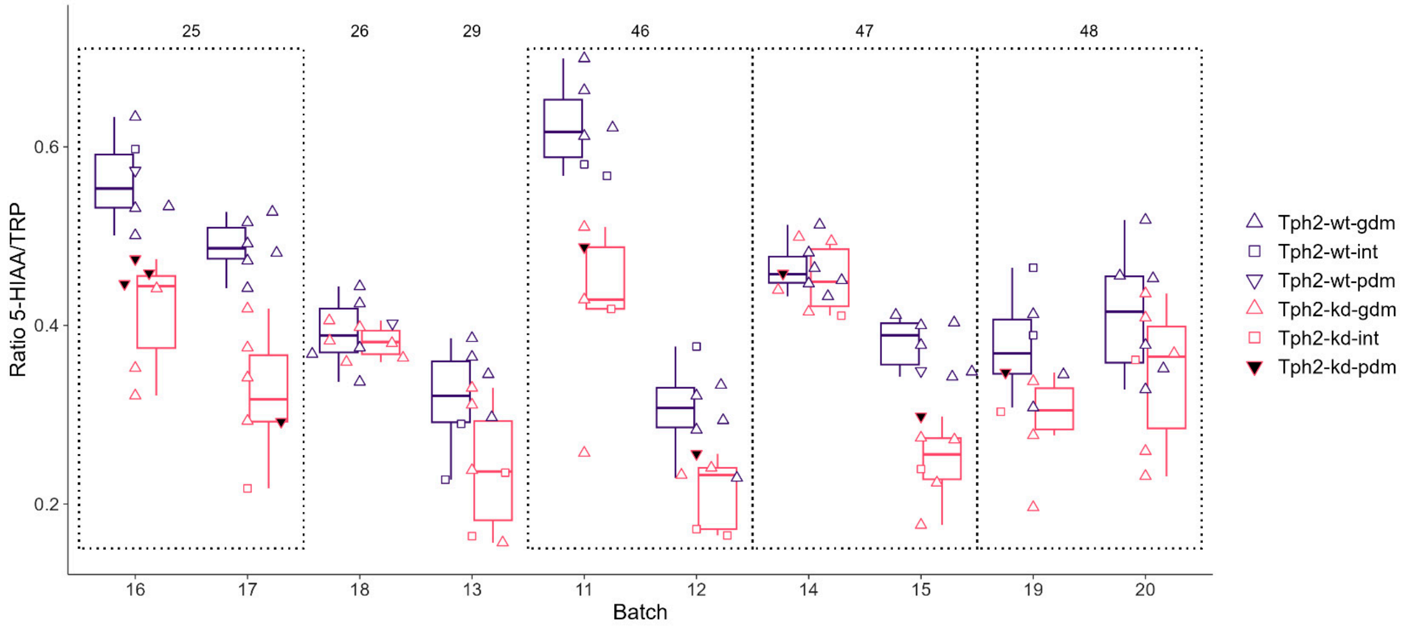

Figure S3. Individual ratios of 5-HIAA and tryptophan. 5-HIAA and tryptophan were more robustly detected with HPLC than 5-HT for all batches. Individual data over boxplots. The duration of treatment in days is indicated at the top of the figure and the dotted lines indicate the batches tested at the same time (pair of batches).
